# Structure and the catalysis mechanism of oxidative chlorination in nanostructural layers of a surface of alumina

**DOI:** 10.1186/1556-276X-9-357

**Published:** 2014-07-15

**Authors:** Sergiy A Kurta, Igor M Mykytyn, Tetiana R Tatarchuk

**Affiliations:** 1 Vasyl Stefanyk Precarpathian National University, Ivano-Frankivsk, Ivano-Frankivsk 76025, Ukraine

**Keywords:** Crystalquasychemical model, Catalyst, Oxidative chlorinating, Reaction, Mass spectrum, Differential, Mechanism

## Abstract

On the basis of X-ray diffraction and mass spectrometric analysis of carrier γ-Al_2_O_3_ and catalysts CuCl_2_/CuCl on its surface, the chemical structure of the active centers of two types oxidative chlorination catalysts applied and permeated type of industrial brands “Harshow” and “MEDС-B” was investigated. On the basis of quantum-mechanical theory of the crystal, field complexes were detected by the presence of CuCl_2_ cation stoichiometry and structure of the proposed model crystal quasichemical industrial catalyst permeated type MEDС-B for oxidative chlorination of ethylene. On the basis of quantum-mechanical calculations, we propose a new mechanism of catalysis crystal quasichemical oxidative chlorination of ethylene reaction for the catalysts of this type (MEDС-B) and confirmed the possibility of such a mechanism after the analysis of mass spectrometric studies of the active phase (H_2_ [CuCl_4_]) catalyst oxidative chlorination of ethylene. The possibility of the formation of atomic and molecular chlorine on the oxidative chlorination of ethylene catalyst surface during Deacon reaction was displaying, which may react with ethylene to produce 1,2-dichloroethane. For the active phase (H [CuCl_2_]), catalyst offered another model of the metal complex catalyst oxidative chlorination of ethylene deposited type (firm ‘Harshow,’ USA) and the mechanism of catalysis of oxidative chlorination of ethylene with this catalyst.

## Background

Determination of the mechanism of catalysis of the process of oxidative chlorination of ethylene to 1,2-dichloroethane (1,2-EDC) gives practical result for their preparation; their use determines the conditions that lead to a decrease in complete oxidation (combustion) of ethylene and reducing the formation of reaction by-products - organo chlorine waste. The aim of the work is to improve the technology of industrial processes of synthesis of 1,2-EDC way oxidative chlorination of ethylene (OCE) as well as improving the technology of this process with a decrease in the amount of organochlorine waste, based on determination of the structure of the reaction centers of catalysts and mechanism of ethylene oxidative chlorination. First, we established effective cooperation mechanism and thermodynamics of heterogeneous reactions of OCE on the structure of surface active groups of the two different types of catalysts Cu (I) (II)/γ-Al_2_O_3_ (presumably deposited and permeated type), their connection with the conversion of raw reagents chloride, ethylene, and oxygen, and an increase in yield 1,2-EDC and fewer by-products formed.

The reaction of the oxidative chlorination gives an opportunity in which it is useful to use НСl, namely, to create the productions balanced on a chlorine, i.e., that does not have wastes of НСl or hydrochloric acid. In industry, 1,2-dichloroethane is obtained in the oxidative chlorination ethylene (EOС) using catalysts based on copper chlorides [1] applied to the surface of the media - γ-Al_2_O_3_. EOС process is carried out in a fluidized bed of catalyst, and a stationary layer at a temperature in the reaction zone is 200 to 250°C and a pressure of 0.25 to 0.4 MPa [2]. It is possible to vary the qualitative and quantitative compositions of OCE products by using different types of CuCl_2_/γ-Al_2_O_3_ catalysts (with copper chlorides supported onto an alumina surface ‘Harshow’ (X1) and located in the internal pores of the support ‘MEDC-B’), and also, the mechanism of process of catalysis is different.

The reaction of OCE in 1,2-EDC proceeds according to Equation (1) [3]:

(1) $${С}_{2}{Н}_{4}+2\mathrm{НСl}+0,5{О}_{2}\to {С}_{2}{Н}_{4}{\mathrm{Сl}}_{2}+{Н}_{2}О+Q$$

Todo et al. [4] believed that the following processes occur with the participation of copper catalysts:

(2) $$2{\mathrm{CuCl}}_{2}+C_{2}H_{4}\to C_{2}H_{4}{\mathrm{Cl}}_{2}+2\mathrm{CuCl}$$

(3) $$2\mathrm{CuCl}+{\mathrm{Cl}}_{2}\to 2{\mathrm{CuCl}}_{2}$$

The chemical interactions between the surface of γ-Al_2_O_3_ support and copper chlorides that were described are not ideal, since copper chlorides are not capable of catalyzing OCE without a support [4]. The description of the mechanism of reactions (2) and (3) [4] without the participation of the surface groups of γ-Al_2_O_3_ is therefore not entirely reliable.

It is believed that the oxygen oxidizes the Cu (I) in oxychlorides Cu (II), which are converted by means of HCl in CuCl_2_ and copper salts as carriers acquire chloro [5] as illustrated in Schemes 5 to 7.

(4) $$\mathrm{Quickly}\;2{\mathrm{CuCl}}_{2}\to \leftarrow 2\mathrm{CuCl}+{\mathrm{Cl}}_{2}$$

(5) $$\mathrm{Slowly}\;2\mathrm{CuCl}+O_{2}\to \leftarrow {\mathrm{Cu}}_{2}O_{2}{\mathrm{Cl}}_{2}$$

(6) $$\begin{array}{l} \mathrm{Quickly}\;{\mathrm{Cu}}_{2}O_{2}{\mathrm{Cl}}_{2}+4\mathrm{HCl}\to \leftarrow 2{\mathrm{CuCl}}_{2}+{\mathrm{Cl}}_{2}\\ \;+2H_{2}O \end{array}$$

However, the mechanism (reactions 7 to 9) submitted by M. Flid and I. Kurlyandskaya [6] is imperfect, as enough specifically - schematically describes the reaction of ethylene with the surface groups of the catalyst during the reaction OCE.

In this article, the new interpretation of description of active centers [СuСl_2_]^-1^, [СuСl_4_]^2-^ on the surface of industrial catalyst γ-Al_2_O_3_ for the process of OCE is offered. On the basis of quantum-mechanical calculations crystalquasichemical model [7], an attempt to explain processes that flow on the surface of [СuСl_2_]^-1^, [СuСl_4_]^2-^/γ-Al_2_O_3_ catalyst is done in the industrial conditions OCE in 1,2-EDC.

## Methods

In order to study copper chloride effects on ethylene oxidative chlorination, five samples were compared as follows:

1. Pure γ-Al_2_O_3_, as a catalyst carrier, firm ‘Harshow’ (USA) *S*_sp_ =140 m^2^ g^-1^; *V*_п_, = 0.4 cm^3^ g^-1^.

2. A commercial catalyst X1 CuCl_2_, applied from muriatic water solution on the γ-Al_2_O_3_ surface, in an amount of 5 wt.% by Cu^+2,+1^, firm ‘Harshow’ (USA) *S*_sp_ = 120 m^2^ g^-1^; *V*_п_, = 0.3 cm^3^ g^-1^.

3. A commercial catalyst MEDС-B based γ-Al_2_O_3_/CuCl_2_ containing of 5 wt.% by Cu^+2,+1^, OXYMAX-B (MEDС-B) firm ‘Sud-Chemie Catalysts’ (Munich, Germany) *S*_sp_ = 140 m^2^ g^-1^; *V*_п_, = 0.36 cm^3^ g^-1^.

4. CuCl_2_ · 2H_2_O - crystalline hydrate of the main component derived from OCE catalysts.

5. CuCl_2_ · 2HСl - hydrochloride of the main component derived from OCE catalysts.

X-ray diffraction patterns were obtained on a DRON-4-07 using the X-ray focus on the Bragg-Brentano. The most suitable for the study was a copper anode radiation (*λ* = 1.54178 Å). The Nі-fіlter was used in reflected light. The velocity of the goniometer is 1 to 2° min^-1^. The sample was prepared by applying a layer of powder on vaseline (amorphous), which had previously been coated with a thin layer on a quartz cuvette.

Mass spectrometry was carried out using monopole mass spectrometer (MX-7304A, AO. SELMI, Sumy, Ukraine, 1 to 210 mass range) with electron impact ionization, converted for thermal desorption measurements. Sample (0.1 to 20 mg) was placed at the bottom of a quartz-molybdenum vessel and pressure was reduced to 5⋅10^-5^ Pa. Samples were heated to 750°С at 0.15°С s^-1^. Volatile products entered the mass spectrometer ionization chamber where they were ionized and fragmented with electron beam. Desorption rate temperature dependence helps identifying thermal transformation products and provides interaction energy between the copper chloride active phase and γ-Al_2_O_3_ carrier [8].

## Results and discussion

### Crystalquasichemical structure of catalyst and mechanism of reaction on its basis

γ-Al_2_O_3_ has a spinel structure. It is known that there are cations vacancies in octahedral positions, which is why crystalquasichemical formula γ-Al_2_O_3_ is written down as:

$${{\mathrm{Al}}^{+3}}_{A}{\left[{\mathrm{Al}}^{+3}{{}_{5/3^{□}}}_{1/3}\right]}_{B}{\left({O^{-2}}_{4}\right)}_{O}$$

where A is tetrahedral positions, B is octahedral positions, □ is crystallochemical vacancy, and O is position of oxygen.

But, crystallchemical composition does not give any information about the active centers on a surface of γ-Al_2_O_3_[9], but a crystalquasichemical model takes into account the presence of donor’s and acceptor’s active centers on the surface of catalyst. Crystalquasichemical composition γ-Al_2_O_3_ can be written down as follows:

$$A1_{A}^{•}{\left[{\mathrm{Al}}_{\frac{5}{3}}^{\times }V_{\frac{1}{3}}^{\prime \prime \prime }\right]}_{B}\;{\left(O_{4}^{\times }\right)}_{O}.$$

where ● is an excess positive charge, ′′′ is a triple excess negative charge, × is an effective zero charge, and V is a vacancy of aluminum in an octahedral sublattice.

Considering that γ-Al_2_O_3_ is the catalyst supports of OCE coated on its surface with CuCl_2_ or Cu_2_Cl_2_, the nature of the catalytic active centers of the catalyst complex is well explained on the basis of crystalquasichemical model. It examines the mechanism of OCE catalysis process, including the formation of cation and anion vacancies in the adsorption and chemisorption of HCl, O_2_, and C_2_H_4_ active centers on the catalyst surface, which act as point defects in the γ-Al_2_O_3_ crystal lattice. CuCl_2_/Cu_2_Cl_2_ will include the structure of γ-Al_2_O_3_, making it defective. In particular, for chemical interaction, CuCl_2_ can be written, given by the stoichiometry cation and anion stoichiometry for:

(a) cation's stoichiometry:

(10) $$\begin{array}{l} \left(1-\alpha \right){\mathrm{Al}}_{A}^{•}{\left[{\mathrm{Al}}_{\frac{5}{3}}^{\times }V_{\frac{1}{3}}^{\prime \prime \prime }\right]}_{B}{\left(O_{4}^{\times }\right)}_{O}+{\mathrm{\alpha Cu}}_{A}^{\times }{\left[{Cu^{\prime }}_{2}\right]}_{B}{\left({\mathrm{Cl}}_{4}^{•}\right)}_{O}{\left(Cl_{2}^{\prime }\right)}_{i}\to \\ \;\to {\left({\mathrm{Al}}_{1-\alpha }^{•}{\mathrm{Cu}}_{\alpha }^{\times }\right)}_{A}{\left[{\mathrm{Al}}_{\frac{5}{3}-\frac{5\alpha }{3}}^{\times }Cu_{2\alpha }^{\prime }V_{\frac{1}{3}-\frac{\alpha }{3}}^{\prime \prime \prime }\right]}_{B}{\left(O_{4-4\alpha }^{\times }{\mathrm{Cl}}_{4\alpha }^{•}\right)}_{O}{\left(Cl_{2}^{\prime }\right)}_{i} \end{array}$$

(b) anion's stoichiometry:

(11) $$\begin{array}{c} \left(1-\beta \right){\mathrm{Al}}_{A}^{•}{\left[{\mathrm{Al}}_{\frac{5}{3}}^{\times }V_{\frac{1}{3}}^{\prime \prime \prime }\right]}_{B}{\left(O_{4}^{\times }\right)}_{O}+{\mathrm{\beta Cu}}_{A}^{\times }{\left[{Cu^{\prime }V}^{\prime \prime \prime }\right]}_{B}{\left({\mathrm{Cl}}_{4}^{•}\right)}_{O}\to .\\ \to {\left({\mathrm{Al}}_{1-\beta }^{•}{\mathrm{Cu}}_{\beta }^{\times }\right)}_{A}{\left[{\mathrm{Al}}_{\frac{5}{3}-\frac{5\beta }{3}}^{\times }Cu_{\beta }^{\prime }V_{\frac{1}{3}+\frac{2\beta }{3}}^{\prime \prime \prime }\right]}_{B}{\left(O_{4-4\beta }^{\times }{\mathrm{Cl}}_{4\beta }^{•}\right)}_{O} \end{array}$$

Thus, on the basis of crystalquasichemical model, it is possible to set nature of active centers of OCE catalyst: chemical interaction of CuCl_2_ with γ-Al_2_O_3_ is accompanied by formation of the engrained ions of Cl^-^ or by the increase of vacancies in octahedral sublattice and also including ions of Сu^2+^, in thetra and octahedral positions of spinel lattice.

According to [10], a decisive influence will do copper chlorides CuCl_2_/Cu_2_Cl_2_ or H_2_ [CuCl_4_], H [CuCl_2_] that are in the crystal structure of spinel lattice on the surface of γ-Al_2_O_3_, after their applying.

Crystalquasichemical model provides two ways of CuCl_2_ representation in spinel structure, considering the stoichiometry by the cations and anions:

$$\begin{array}{l} Cu_{A}^{\times }{\left[Cu_{2}^{\prime }\right]}_{B}{\left(Cl_{4}^{•}\right)}_{O}{\left(Cl_{2}^{\prime }\right)}_{i}\;\mathrm{and}\;Cu_{A}^{\times }{\left[Cu_{2}^{\prime }V^{\prime \prime \prime }\right]}_{B}{\left(Cl_{4}^{•}\right)}_{O}\\ \;\left(I\right)\;\;\left(\mathrm{II}\right) \end{array}$$

The presence of vacancies or root defects in the crystal structure is confirmed by determination of a pycnometer density of CuCl_2_ sample. In this study, the theoretical X-ray density of CuCl_2_ (*ρ*_theor._ = 3,438.39 kg m^-3^) is less than the practical density of CuCl_2_; we found *ρ*_prakt._ = 3,773.61 kg m^-3^ that indicates the presence of root defects in the CuCl_2_ structure (formula I).

Adsorption and chemical processes in the catalytic OCE will pass through the formation of cation and anion vacancies in the spinel structure of the catalyst.

In accordance with a crystal quasichemical mechanism, to our opinion, the first stage of process will be adsorption of gaseous НСl on the active centers of surface of γ-Al_2_O_3_ catalyst with coated CuCl_2_. The gaseous molecules of HCl adsorbed on the catalyst surface (a chlorine atom in the position of oxygen $Cl_{О}^{•}$ and copper atoms in the octahedral sublattice), contributing to the formation of anionic vacancies in the oxygen sublattice. Polar molecule HCl held on the catalyst surface by Van der Waals interaction forces. Based on the recorded crystal quasichemical formula, surface interaction active centers appear atoms having excess charge, which is copper atoms into octahedral sublattice ${\left[Cu_{2}^{\prime }\right]}_{B}$ and the atoms of chlorine ${\left(Cl_{4}^{•}\right)}_{O}$ in the positions of oxygen. Given that the equilibrium internuclear distance *r*_0_ in the molecule of hydrogen chloride is equal to 0.127 nm and a molecular diameter HCl 0.3 nm, respectively, hydrogen atom occupancy crystalquasichemical pattern recorded in the tetra and octahedral positions of the spinel structure. The adsorbed hydrogen will react with oxygen to form water molecules (the hydrogen must be twice thе oxygen), so that when writing the crystal quasichemical equations for the formation 4α mole H_2_O use 8α mole of HCl and 2α mole of gaseous O_2_. Given that cupric chloride catalyst performs matrix and adsorbed HCl impurity, the amount of matrix and impurity to form an impurity cluster should be 1 mole. So, crystal quasichemical equation introduces the following factors:

Thus, the absorption reaction of hydrogen chloride on the surface of the cupric chloride catalyst is accompanied by the formation of defects anion vacancies ${\left(V_{8\alpha /3}^{••}\right)}_{O}$ in the position of the oxygen and hydrogen in the placement of tetrahedral and octahedral positions ${\left(H_{8\alpha /3}^{\prime }\right)}_{A}\;и\;{\left[H_{16\alpha /3}^{\prime \prime }\right]}_{B}$.

Oxygen, as an electron acceptor, being adsorbed on the surface of spinel catalyst, predetermines the origin of acceptor type.

Thus, in a spinel structure, cationic vacancies occur in the tetrahedral (A) and octahedral (B) sublattices:

(13) $$\begin{array}{l} \left(1-\alpha \right){\mathrm{Cu}}_{A}^{\times }{\left[{Cu^{\prime }}_{2}\right]}_{B}{\left({\mathrm{Cl}}_{4}^{•}\right)}_{O}{\left({Cl^{\prime }}_{2}\right)}_{i}{{+2\mathrm{\alpha O}}_{2}}_{\left(\mathrm{gas}\right)}\\ \;+\alpha \underset{\mathrm{spinel}\;\mathrm{antistructure}}{\underbrace{V_{A}^{\prime \prime }{\left[V_{2}^{\prime \prime \prime }\right]}_{B}{\left(V_{4}^{••}\right)}_{O}}}\\ \to \to {\left({\mathrm{Cu}}_{1-\alpha }^{\times }V_{\alpha }^{\prime \prime }\right)}_{A}{\left[Cu_{2-2\alpha }^{\prime }V_{2\alpha }^{\prime \prime \prime }\right]}_{B}{\left(O_{4\alpha }^{\times }{\mathrm{Cl}}_{4-4\alpha }^{•}\right)}_{O}\\ \;{\left({Cl^{\prime }}_{2-2\alpha }\right)}_{i}+8{\mathrm{\alpha h}}^{•} \end{array}$$

This adsorbed hydrogen reacts with the oxygen in the crystalline lattice of the spinel formation molecules H_2_O:

(14) $$\begin{array}{l} 4\alpha O_{O}^{\times }+\frac{8}{3}\alpha H_{A}^{\prime }+\frac{16}{3}\alpha H_{B}^{\prime \prime }\to 4\alpha H_{2}O+\frac{8}{3}\alpha V_{A}^{\prime \prime }\\ \;+\frac{16}{3}\alpha V_{B}^{\prime \prime \prime } \end{array}$$

On-site oxygen atoms that have left the crystal lattice of defect catalyst anion vacancies remained as follows:

(15) $$\begin{array}{l} 4\alpha O_{O}^{\times }\to 4\alpha O^{‒2}+4\alpha V_{O}^{••}+8\alpha e^{\prime }\\ \;↓\\ \;4\alpha {Н}_{2}О \end{array}$$

It can be seen that as a result of this interaction, electronic conductivity and vacancies in the anion sublattice appear (oxygen of the lattice involved in the oxidation reaction).

Adsorbed chlorine atoms ${\left(Cl_{4‒8\alpha /3}^{•}\right)}_{O}$, which were formed as a result of the above processes, here, react with molecules adsorbed ethylene by their adherence to the double bond with the formation of 1,2-EDC:

(16) $$\begin{array}{ll} \left(4-\frac{8}{3}\alpha \right){\mathrm{Cl}}_{О}^{\times } & +\left(2-\frac{4}{3}\alpha \right)C_{2}H_{4}\to \left(2-\frac{4}{3}\alpha \right)C_{2}H_{4}{\mathrm{Cl}}_{2}\\ & +\left(4-\frac{8}{3}\alpha \right)V_{O}^{\times } \end{array}$$

The next stage of the mechanism of catalysis is the annihilation (disappearance) of antistructure spinel and hole-electron pairs, which returns to the original state of the catalyst:

(17) $$\frac{8}{3}\alpha V_{A}^{\prime \prime }+\frac{16}{3}\alpha V_{B}^{\prime \prime \prime }+\frac{32}{3}{\mathrm{\alpha V}}_{{}^{O}}^{••}\to \frac{8}{3}\underset{\mathrm{annigilation}}{\underbrace{\alpha V_{A}^{\prime \prime }{\left[V_{2}^{\prime \prime \prime }\right]}_{B}{\left(V_{4}^{••}\right)}_{O}}}$$

(18) $$\alpha V_{A}^{\prime \prime }+2\alpha V_{B}^{\prime \prime \prime }+4{\mathrm{\alpha V}}_{O}^{••}\to \underset{\mathrm{annigilation}}{\underbrace{\alpha V_{A}^{\prime \prime }{\left[V_{2}^{\prime \prime \prime }\right]}_{B}{\left(V_{4}^{••}\right)}_{O}}}$$

(19) $$\underset{\mathrm{annigilation}}{\underbrace{8{\mathrm{\alpha h}}^{•}+8\alpha e^{\prime }}}=0\left(\mathrm{zero}\right)$$

Thus, adsorption of gaseous HCl and O_2_ gives rise to defects - cationic and anionic vacancies. OCE processes on the catalyst permeated type MEDС-B through a defective condition of CuCl_2_, which then passes from the initial state that is fully consistent theory of catalysts.

### X-ray phase analysis of investigational standards of carrier, catalysts and chlorides of copper

For more detailed data investigations of carrier, catalysts and active phase of CuCl_2_ were conducted using the X-ray analysis.

**Figure 1:**
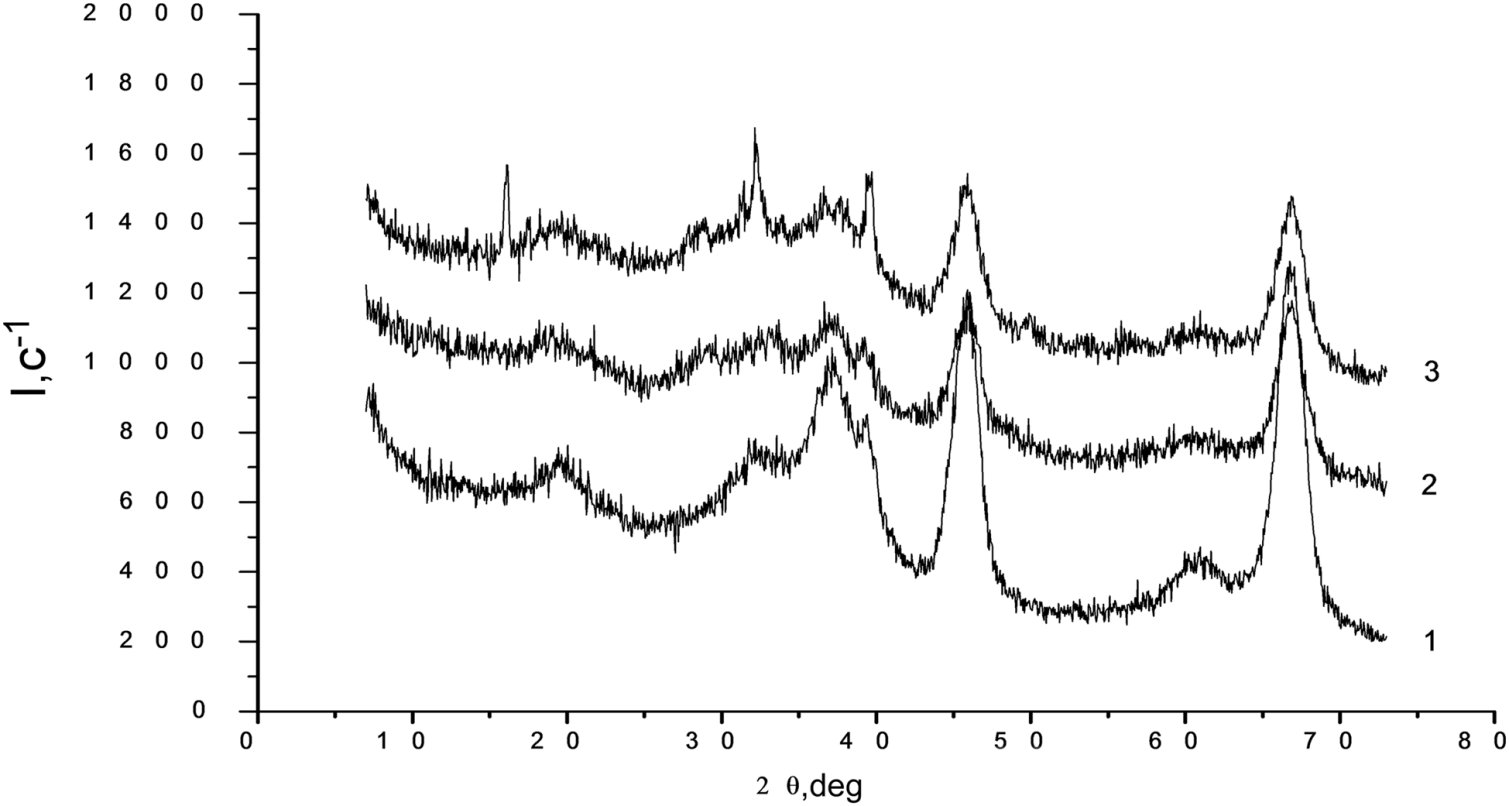
**Diffraction patterns γ-Аl**_**2**_**О**_**3 **_**(1), X1 catalysts as received (2) and after a few days of air exposure (3).**

**Figure 2:**
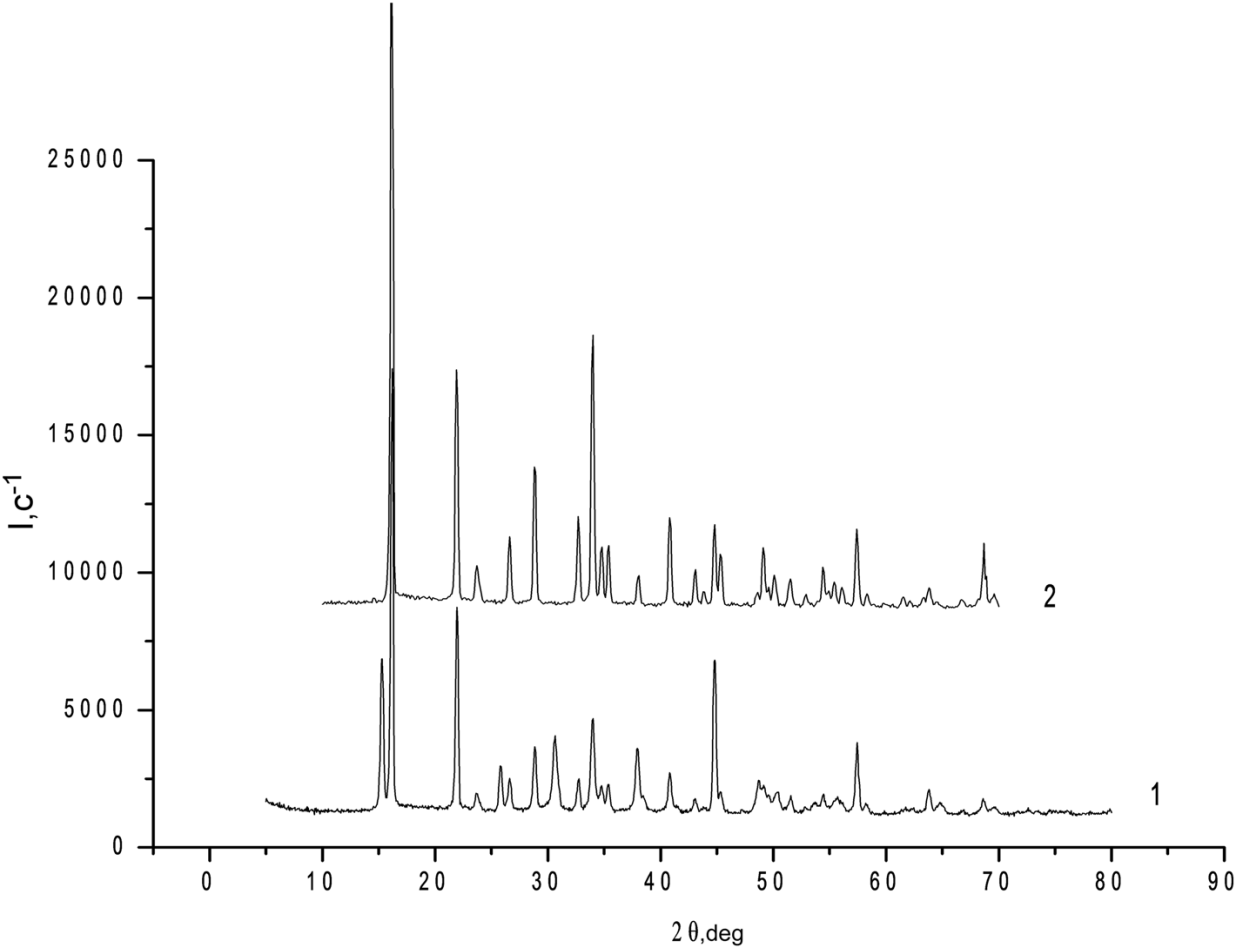
**Diffraction patterns of CuCl**_**2 **_**2HСl or H**_**2 **_**[СuСl**_**4**_**] main catalyst components before (1) and after a few days of air exposure (2).**

Diffraction patterns of catalyst samples X1 have differences from the original diffraction pattern of the sample γ-Al_2_O_3_ (Figure 1). These differences consist in reducing the intensity of the diffraction pattern peaks by 25 to 27% at 2θ in the range of 66.9°, 45.9°, and 36.65°. This indicates the presence of other phases in the sample [CuCl_2_]^-1^ and [CuCl_4_]^-2^, which interact with the surface groups of Al_2_O_3_ and reduce their intensity. Furthermore, on the X1 catalyst sample curve, an additional new peak maximum at 28.55° (Figure 1) is found. It can be identified as a new phase of pure CuCl_2_[7]; in this case, indicated peaks do not coincide with peaks on the diffraction patterns of pure copper chloride CuCl_2_ (Figure 2). Prосеeding from it can be said that cuprum chloride, which is applied on to the surface of γ- Al_2_O_3_, interact in adsorption way with hydroxy- and alumoxy-surface groups . Therewith, pure catalyst X1 as it is seen in diffractograms comparison (curves 2, 3, Figure 1) does not contain copper hydrochlorides or contains it in a very small amount, which is corresponded to peaks of 32.4°, 36.65°, and 39.25°. When X1 catalyst exists in the air conditions on diffractograms of the catalyst, an additional peak appears at 16.1°, which is not a pu*r*e sample of the X-ray (Figure 1). Obviously, this can be attributed to the formation in the air conditions (presence of water vapor) of mixed copper hydroxides, hydroxychlorides Cu(OH)Cl or Cu(OH)_2_ and CuCl_2_•2H_2_O (Figure 2) [3].

**Figure 3:**
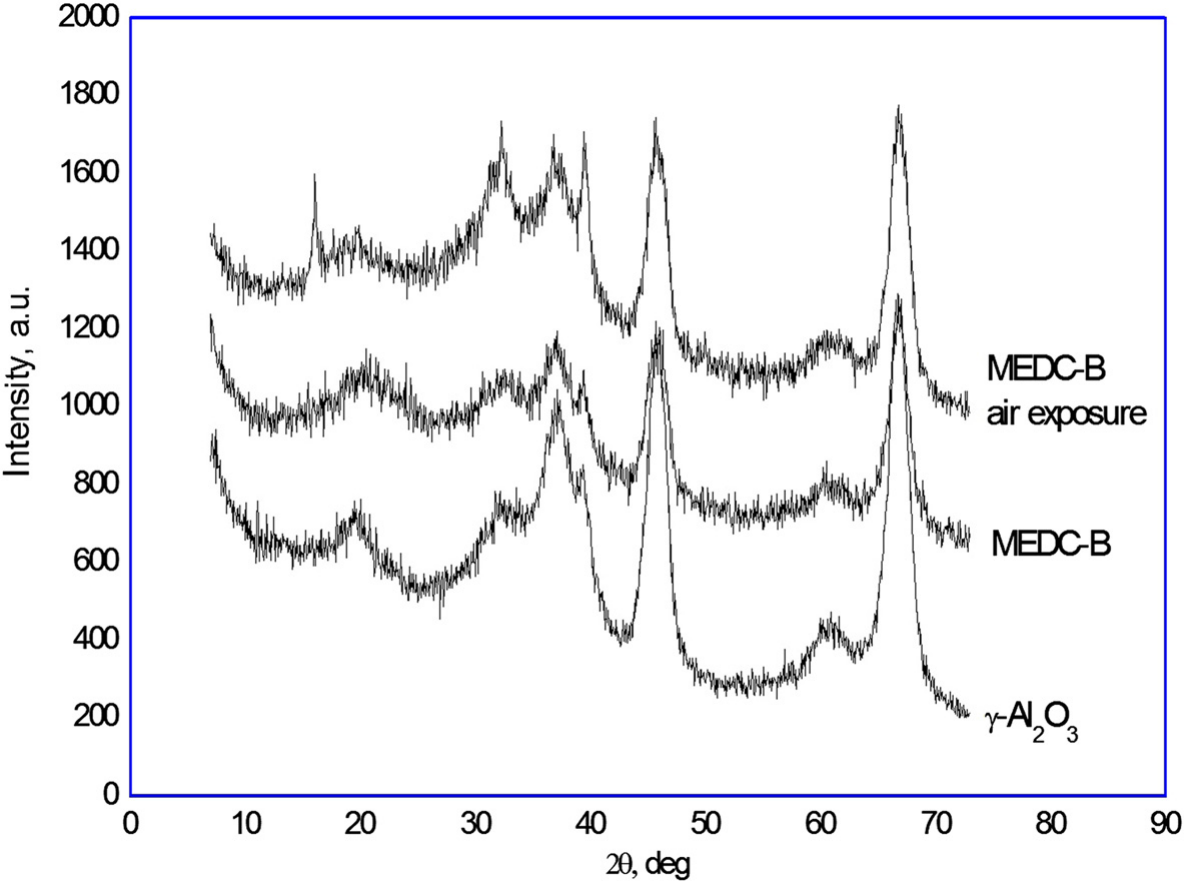
**Diffraction patterns of γ-Аl**_**2**_**О**_**3**_**, MEDC**-**B catalysts as**-**received and after a few days of air exposure.**

MEDC-B catalyst diffraction pattern (Figure 3) suggests that as with the X1 catalyst, copper chloride application onto the γ-Аl_2_О_3_ surface also proportionally decreases 37.1°, 45.75°, and 67° 2θ reflections intensity by 25%. Such intensity decrease indicates the presence of [CuCl_2_]^-^, [CuCl_4_]^2-^, and Cu(OH)Cl phases in the MEDC-B catalyst which interact with Аl_2_О_3_ surface groups.

In contrast to the X1 catalyst, there is no crystalline CuCl_2_ reflection at 28.55° for the MEDC-B catalyst (Figure 1). This leads to the conclusion that all active copper chloride is evenly distributed throughout all MEDC-B catalyst particles volume and bound to the Аl_2_О_3_ surface.

From the diffraction data, it is clear that the structure of the CuCl_2_ catalyst surface compounds on the γ-Аl_2_О_3_ carrier is different for the X1 and MEDC-B catalysts. At the same time, X1 catalyst 32°, 37°, and 39° intensities differ greatly. This indicates the presence of at least three, and possibly more different compounds between CuCl_2_ and γ-Аl_2_О_3_ on the X1 catalyst surface confirmed by the literature sources [11]. Based on the difference spacings (Δd) of the catalyst carrier d (Al_2_O_3_) and catalysts X1 (d_X1_) and MEDC-B (d_MEDC-B_), the following conclusions can be drawing.

X1 catalyst , if compared to the γ-Аl_2_О_3_ carrier, is characterized by the inter-planar spacing increase for the characteristic 28.55°, 36.55°, 39.25°, 45.9°, and 79.7° diffraction lines, which is an indication of a wedging effect of the excess pure CuCl_2_ + CuCl crystal phase [11].

In contrast, MEDC-B catalyst (Table 1), if compared to the γ-Аl_2_О_3_ carrier, is characterized by the inter-planar spacing decrease for 20.55°, 32.6°, 39.45°, and 68.0° diffraction lines, which is an indication of homogeneity of phases of the catalyst and the carrier, in which the catalyst is present both on the surface and in the structure without CuCl_2_ crystal phase.

**Table 1:** **The angular position of the diffraction lines**, **2θ corresponding distances between planar**, **d**, **and the relative intensities**, **I**/**I**_**0**_, **sample** “**MEDC**-**B**”

**Number**	**2θ, °**	**Intensities І, С**^**-1**^	**d, А**	**I/I**_**0**_**, %**	**Δd = d**_**MEDС-B**_**-dAl**_**2**_**O**_**3**_
1	16.95	1,092	5.23071	73.39901	-
2	20.55	1,150	4.32179	82.92282	-0.1066
3	23.95	1,111	3.71541	76.51888	-
4	32.6	1,098	2.74664	74.38424	-0.0332
5	37.1	1,193	2.42318	89.98358	+0.0031
6	39.45	1,094	2.28408	73.72742	-0.0055
7	45.75	1,219	1.98314	94.25287	+0.00122
8	60.5	860	1.53023	35.30378	-
9	67	1,254	1.3967	100	-0.0046

### Mass spectrometry of investigational standards of copper chlorides

X1 and the MEDC-B catalysts, the γ-Аl_2_О_3_ carrier, active CuCl_2_ · Н_2_О, and CuCl_2_ · 2HСl or Н_2_[CuCl_4_] phases were analyzed using temperature-programmed desorption mass spectrometry to identify individual thermal transformation products on the catalyst surface, the carrier, and the copper chloride active phase.

**Figure 4:**
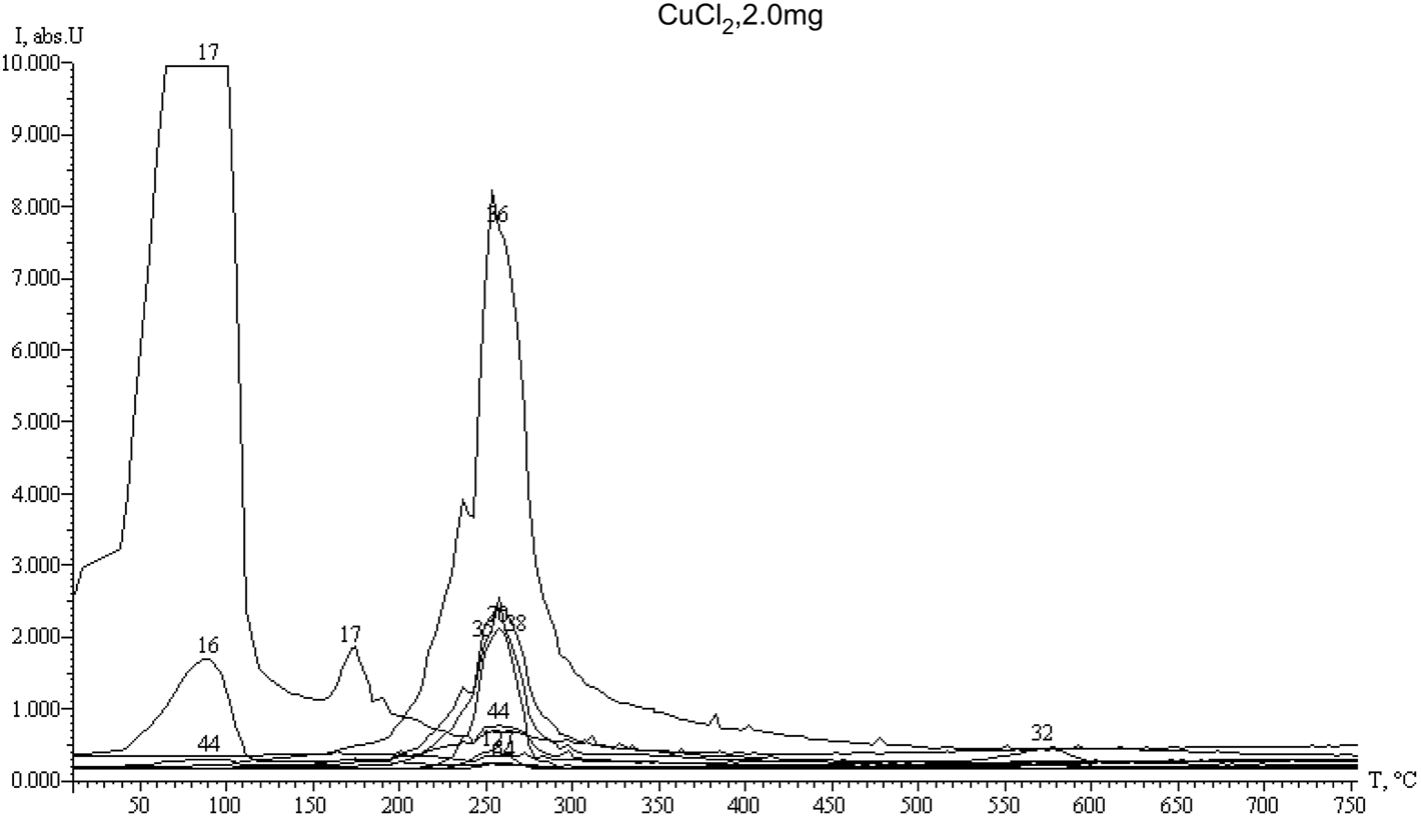
**Thermal desorption curves of the CuCl**_**2 **_**· Н**_**2**_**О active phase decomposition.**

**Figure 5:**
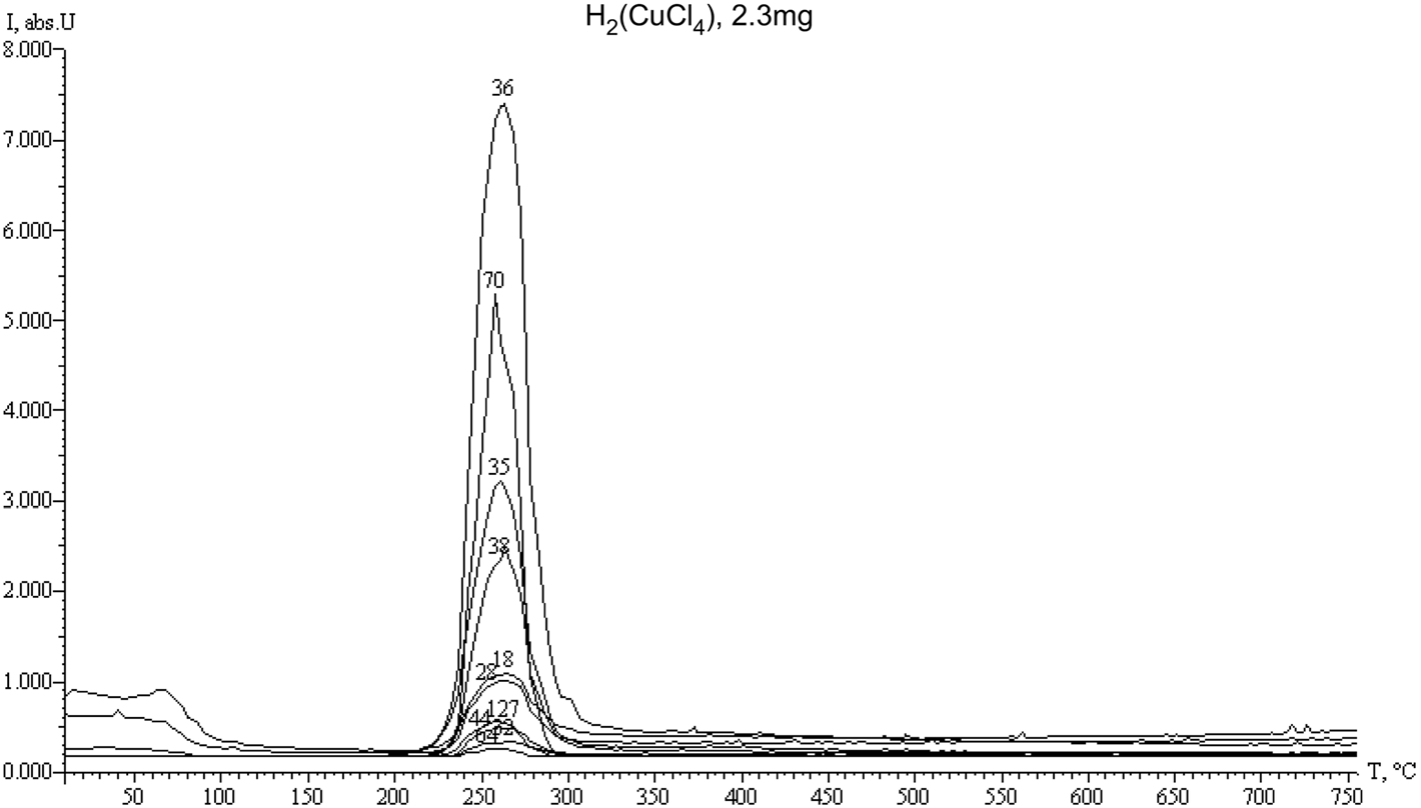
**Thermal desorption curves of the CuCl**_**2**_** · 2HСl or Н**_**2**_**[CuCl**_**4**_**] active phase decomposition.**

Desorption curve analysis of the active CuCl_2_ · Н_2_О phase (Figure 4) and CuCl_2_ · 2HСl or Н_2_[CuCl_4_] phases (Figure 5) suggests the following. In the 100 to 200°C temperature range, partially hydrolyzed CuCl_2_ · Н_2_О active phase looses large amounts of adsorbed and structured water. Internally, crystallized water release starts at 170°C, while adsorbed water release starts above 50°C, which is not observed with a pure CuCl_2_ · 2HСl or Н_2_ [CuCl_4_] active phase. Intensive dehydrochlorination processes of the CuCl_2_ · Н_2_О active phase occur in the 200 to 300°С range identified by maximum HCl desorption peaks at 260°С for both types of active phases. However, with pure non-hydrolyzed CuCl_2_ · 2HСl or Н_2_ [CuCl_4_] phases dehydrochlorination processes intensity is higher and consists of three peaks, whereas with a partially hydrolyzed CuCl_2_ · Н_2_О, it consists of only two HCl desorption peaks (M_HCl_ = 35 to 38). Still, the most interesting characteristic of the active phase decomposition products is the intense molecular chlorine Cl_2_ desorption peak (М_Cl2_ = 70), which is two times more intense with a pure active Н_2_ [CuCl_4_] phase. This phenomenon has not been observed before with other analysis methods and provides evidence of the active phase decomposition reaction with Cl_2_ release (reaction 16), which may be involved in ethylene chlorination reactions 21–22.

The similar phenomena of selection of chlorine from the active phase of catalysts are known from literature at transformation of copper chloride to the monochloride of copper [12].

(20) $$\begin{array}{l} \;\\ {Н}_{2}\left[{\mathrm{CuCl}}_{4}]\;\overset{220˚C}{\to }{Н}_{2}[{\mathrm{CuCl}}_{2}\right]+\;1/2{\mathrm{Сl}}_{2}+\;\mathrm{НСl} \end{array}$$

(21) $$H_{2}\left[\mathrm{CuC}l_{4}\right]\overset{235˚C}{\to }2\mathrm{HCl}+\mathrm{CuC}l_{2}$$

(22) $$2\mathrm{CuC}l_{2}=2\mathrm{CuCl}+Cl_{2}$$

Taking into account aforesaid, it is possible to speak about confirmation of structure of active centers on the surface of Х1 and MEDC-B catalysts [7]. Besides, HCl and Сl_2_ release reactions 20 to 22 in the processes of the active phase thermal destruction at the 210 to 235°C working temperature range which confirms the OCE reaction crystalquasichemical mechanism described in reactions 12 to 19 and presented below.

### Prognostic mechanism of OCE reactions on the surface of catalysts

From the other side in the previous publications [13] we described, that the formation reactions of metal-complex compounds can run between the superficial groups of carrier and catalyst. On the surface of catalyst of the ethylene oxidized chlorination, three types of complexes between aquated Al_2_О_3_ and complex compounds of copper [СuСl_4_]^2-^, [СuСl_2_]^-^ і [СuСl_4_]^-^[14] are formed.

According to the data of IR spectral analysis of catalysts with various structures MEDC-B and X1, it can be said [15] that the efficiency of the interaction between carrier and CuCl_2_/Cu_2_Cl_2_ and γ-Al_2_O_3_ increases when going over from the applied catalyst X1 type to permeated catalyst type - MEDC-B. This is caused by the technology of their preparation. On this basis, it can be concluded that the preparation technology of MEDC-B catalyst differs from the preparation technology of for type catalyst deposited - X1. Synthesis of microparticles of catalyst [CuCl_2_]^-^, [CuCl_4_]^2-^ and a carrier γ-Al_2_O_3_ with the size of 20 to 120 microns leads to more intense interaction between them and to the formation of intercalation active adsorption centers on the surface and in the pores of the catalyst structure MEDC-B (reaction 10). At the same time, the simple application of CuCl_2_ from chloride solution on the surface of solids γ-Al_2_O_3_ (brand X1) the usual adsorption [CuCl_4_]^2-^, [СuСl_4_]^-^ only on the surface of γ-Al_2_O_3_ runs. As a result of the interaction surface, only partially coordination bonds between the carrier and the catalyst are formed [13].

We have noticed [16] that the use of catalysts such as applied-X1 due time (1 to 2 years) takes a significant (50%) loss of catalyst [СuСl_4_]^2-^, [СuСl_4_]^-^ from the surface of the support γ-Al_2_O_3_ at its mechanical destruction (erase). It leads to a significant reduction in the effectiveness of the catalyst in the oxidative chlorination of ethylene into 1,2-dichloroethane and requires regeneration [17]. In contrast to, the permeated catalyst type MEDC-B runs 3 to 4 years, essentially, without loss of activity, thus, it can be taken out from the process, store several years, and continue to run the process without losing their efficiency [18]. This directly shows the difference in the structure of different types of catalysts.

Based on the X-ray phase analysis and earlier presented electron microscopy results [19], infrared spectroscopy and differential thermal analysis of the Аl_2_O_3_ carrier samples with Х1 and MEDC-B catalysts, the following process is suggested.

For reactions 23 to 24 *m*, *n* = 1 to 2.

For reactions 25 to 26 *m*, *n* = 1 to 4.

In [20], the work expresses the idea of coordinating the interaction of copper with KCl and HCl, at first slow stage of oxygen adsorption, which should lead to a decrease in the effective charge of the cation of copper and lower thermodynamic limitations inherent CuСl_2_ reaction with oxygen [1]. This agrees with the data to reduce the work function of an electron in the transition from pure CuСl_2_ to mixtures with KCl [21]. From this, we can predict metal-complex mechanism of catalysis of OCE reaction on the chart by reactions 23 to 26.

Besides, in the practice of industrial use, it is known [18] that on the coated catalysts (type X1), the ratio of basic reagents OСE reaction of НCl:С_2_Н_4_:О_2_ is (1.9 to 2):(1):(0.7 to 0.8). At the same time, in the permeated type of catalysts, this ratio increases toward increasing of hydrogen chloride and ethylene and oxygen reduction - НCl:С_2_Н_4_:О_2_ as (2 to 2.2):(1 to 1.2):(0.5 to 0.6) [16]. This promotes an increase in productivity of the main reaction for producing 1,2-EDC, based on the unit area of the catalyst, and is possible only if OСE catalysts [22] have a difference in the structure as shown above. Also previously [23], we pitverdyly difference in the two types of catalyst. So, at almost the same operating conditions (*t* ≈ 211°C, 50% load), combustion catalyst for ethylene X1 is 2.45 times more than the catalyst MEDC-B, etc. Based on [22], these studies concluded that the load on the reactor in which the catalyst MEDC-B can be supported in maximum (twofold greater than the reactor in which the catalyst type X1) without any loss of quality indicators response. As the selectivity increases, which will lead to an increase in the productivity of the entire process OHE.

## Conclusions

The following conclusions can be drawn.

1. The structure of ([СuСl_4_]^-2^, [СuСl_2_]^-1^) active catalyst centers used for ethylene oxidative chlorination on the γ-Аl_2_О_3_ carrier was described, namely, two types of catalysts were considered as follows: deposited X1 catalyst and permeated MEDC-B catalyst. It is shown that between CuCl_2_ and superficial groups, γ-Al_2_O_3_ (≡Аl-ОН) passes formation of complex connections with [СuСl_4_]^-2^, [СuСl_2_]^-1^ for the catalyst of the inflicted type of Х1 and for the catalyst of the permeated type of CuCl_2_/Cu_2_Cl_2_, there will be intercalation in a structure γ-Al_2_O_3_, creating in her defects of the stoichiometry after cation.

2. The new model crystalquasichemical mechanism of catalysis of the ethylene oxidative chlorination reaction into 1,2-dichlorethane was proposed, which can be applied for the explanation of catalysis on the surface of industrial catalyst of the permeated with type of -МЕДС-В that is confirmed by experimental data. The mechanism of catalysis of OCE in 1,2-EDC on the industrial catalysts of the inflicted type of -Х1 well explains the metal-complex approach offered and described by us before.

## Competing interests

The authors declare that they have no competing interests.

## Authors' contributions

SAK described the structure of ([СuСl_4_]^-2^, [СuСl_2_]^-1^) active catalyst centers used for ethylene oxidative chlorination on the γ-Аl_2_О_3_ of deposited X1 catalyst and the mechanism of catalysis of oxidative chlorination of ethylene with this catalyst was proposed, and conceived the study and participated in its design and coordination. IMM participated in the design of the study, performed the statistical analysis and drafted the manuscript, and carried X-ray diffraction and mass spectrometric analysis. TRT described crystalloquasychemical composition of γ-Al_2_O_3_ and the new model crystalquasichemical mechanism of catalysis of the ethylene oxidative chlorination reaction into 1,2-dichlorethane was proposed and explained the nature of the active centers of the surface of carrier γ-Al_2_O_3_ and catalysts CuCl_2_/CuCl on its surface. All authors read and approved the final manuscript.
